# Expression levels of insulin-like growth factors and receptors in hepatocellular carcinoma: a retrospective study

**DOI:** 10.1186/1477-7819-12-231

**Published:** 2014-07-22

**Authors:** Yun Shin Chun, Min Huang, Lori Rink, Margaret Von Mehren

**Affiliations:** 1 Hepato-Pancreato-Biliary Programs, Virginia Piper Cancer Institute, 800 E. 28th Street, Suite 602, Minneapolis, MN 55407, USA; 2 Department of Pathology, Fox Chase Cancer Center, 333 Cottman Avenue, Philadelphia, PA 19111, USA; 3 Department of Medical Oncology, Fox Chase Cancer Center, 333 Cottman Avenue, Philadelphia, PA 19111, USA

**Keywords:** hepatocellular carcinoma, insulin-like growth factor, nonalcoholic fatty liver disease

## Abstract

**Background:**

The insulin-like growth factor (IGF) pathway is implicated in the pathogenesis of hepatocellular carcinoma (HCC) and may be important in nonalcoholic fatty liver disease (NAFLD). The aim of this study is to determine expression levels of IGFs and receptors in NAFLD-associated HCC.

**Methods:**

Tissue microarrays were constructed from patients who underwent hepatectomy for HCC. Immunohistochemistry was performed using antibodies for IGF ligands and receptors. Immunostain results were scored by a pathologist blinded to clinical data.

**Results:**

Among 27 patients with HCC, the most common underlying liver diseases included NAFLD, hepatitis C, and alcoholic hepatitis. Expression levels of IGFs and receptors were not associated with patients’ underlying liver disease. In all patients, IGF-2 expression was upregulated in tumor and adjacent non-neoplastic liver. Expression of IGF-1 was low in adjacent liver in 6 of 10 patients with cirrhosis, compared with 2 of 17 patients without cirrhosis (*P* = 0.025). Higher IGF-1 expression in liver adjacent to tumor was associated with poorer median survival of 22 months, compared with 72 months with equal or lower IGF-1 expression in adjacent liver relative to tumor (*P* = 0.006).

**Conclusions:**

Our preliminary results demonstrate significant associations between IGF-1 expression and liver cirrhosis and survival after resection in patients with HCC, independent of their underlying liver disease.

## Background

Hepatocellular carcinoma (HCC) is the fifth most prevalent cancer worldwide and the fastest growing cause of cancer-related death in the United States male population [1]. Nonalcoholic fatty liver disease (NAFLD) is increasingly recognized as a growing cause of nonalcoholic steatohepatitis, progressing to cirrhosis and HCC. Alarmingly, NAFLD may directly promote hepatocarcinogenesis independent of steatohepatitis or cirrhosis [2]. In addition, NAFLD may exert synergistic effects with viral hepatitis in the development of HCC [3]. Nonalcoholic fatty liver disease is the major hepatic manifestation of the metabolic syndrome, marked by obesity, type 2 diabetes, hypertension, or hyperlipidemia. Population studies demonstrate a 90% increased risk of HCC in the obese, while type 2 diabetes confers a three-fold increased risk [4,5]. Both NAFLD and associated HCC are growing public health concerns, owing to the epidemic of obesity in the United States, with nearly two-thirds of the adult population classified as overweight or obese [6,7].

The molecular basis for the transformation of NAFLD into HCC is poorly understood. Previous studies have demonstrated that the insulin-like growth factor (IGF) signal transduction pathway is activated early in up to 90% of HCC and may be particularly important in patients with the metabolic syndrome and NAFLD [8]. The hallmark of the metabolic syndrome is insulin resistance, and the resultant hyperinsulinemia leads to decreased concentrations of IGF binding proteins and increased levels of bioavailable insulin-like growth factor-1 (IGF-1) [9]. In addition, plasma levels of insulin-like growth factor-2 (IGF-2) are increased in obese and type 2 diabetic patients [10]. Signaling through the insulin and IGF-1 receptors has been shown to increase cellular proliferation, inhibit apoptosis, and promote metastasis.

**Figure 1:**
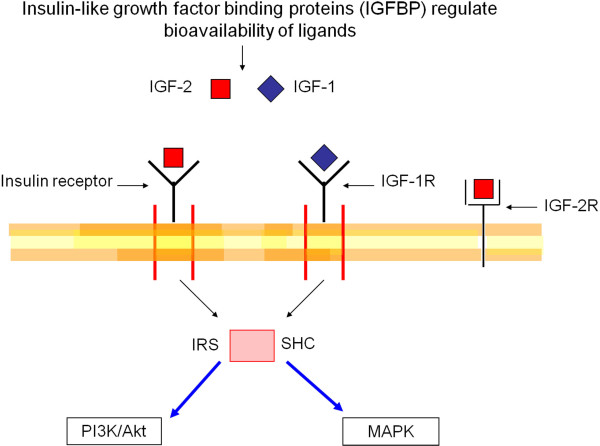
**Insulin-like growth factor pathway.** Bioavailability of the ligands IGF-1 and IGF-2 is regulated by IGF binding proteins. The insulin receptor and IGF-1R are tyrosine kinase cell-surface receptors that recruit and phosphorylate adaptor proteins belonging to the insulin receptor substrate or SHC family, leading to activation of the PI3K/Akt and MAPK signaling cascades. IGF-2R lacks an intracellular tyrosine kinase domain and leads to IGF-2 degradation. IGF-1, insulin-like growth factor-1; IGF-1R, insulin-like growth factor-1 receptor; IGF-2, insulin-like growth factor-2; IGF-2R, insulin-like growth factor-2 receptor; IRS, insulin receptor substrate; MAPK, mitogen-activated protein kinase; P13K/Akt, phosphatidylinositide 3-kinase/Akt; SHC, Src homology 2 domain-containing transforming protein 1.

The IGF pathway is composed of ligands, receptors, substrates, and ligand binding proteins (Figure 1) [11,12]. The ligands are insulin, IGF-1, and IGF-2. Insulin-like growth factor-1, produced mainly in the liver, circulates as an endocrine hormone but is also synthesized in target tissues, where it works in a paracrine or autocrine fashion. Insulin-like growth factor-2 is highly expressed in fetal liver but rapidly downregulated after birth, and its physiologic function in adult liver remains obscure. The IGF-1 receptor (IGF-1R) is a tyrosine kinase cell-surface receptor that is activated by IGF-1 and IGF-2, and by insulin at a much lower affinity. Signaling via IGF-1R leads to phosphorylation of insulin receptor substrate and Src homology 2 domain-containing transforming protein 1 (SHC). Phosphorylation of these receptor substrates results in activation of the phosphatidylinositide 3-kinase/Akt (PI3K/Akt) and mitogen-activated protein kinase (MAPK) signaling cascades and plays a key role in promoting malignant transformation of many cancers, including breast, colon, and lung cancer. The insulin-like growth factor-2 receptor (IGF-2R) lacks a tyrosine kinase domain and acts as a growth inhibitor by directing IGF-2 to lysosomal degradation. This receptor has low affinity for IGF-1 and does not bind insulin [13].

This study sought to determine expression levels of IGFs and their receptors in patients with HCC associated with NAFLD and other underlying liver diseases. We hypothesized that patients with NAFLD-related HCC have alterations in the IGF pathway that are distinct from those in patients with HCC from other causes, such as viral and alcoholic hepatitis. Identification of upregulated components of the IGF pathway may have potential therapeutic implications for patients who may benefit from treatment with IGF-1R inhibitors, which have shown promising anticancer activity in clinical trials [11].

## Methods

### Tissue microarray construction

Formalin-fixed, paraffin-embedded blocks from patients who underwent liver resection for HCC at Fox Chase Cancer Center (Philadelphia, PA, USA) were used to construct tissue microarrays. Areas containing viable tumor cells and adjacent non-neoplastic liver parenchyma >1 cm from the tumor margin were marked by a pathologist. Three 1.0-mm cores were taken from tumor and non-neoplastic tissue and arrayed on a paraffin block.

### Immunohistochemistry

Immunohistochemistry was performed as previously described [14,15]. Briefly, after deparaffinization and rehydration, tissue sections (5 μm thick) were subjected to antigen retrieval, followed by peroxidase block and treatment with a blocking reagent for nonspecific binding. The slides were then incubated overnight with primary antibody at 4°C in a humidified chamber. The following primary antibodies were used: IGF-1 (Santa Cruz Biotechnology, Santa Cruz, CA, dilution 1:100), IGF-2 (Abcam, Cambridge, MA, 1:500), IGF-1R (Cell Signaling, Danvers, MA, 1:50), and IGF-2R (Cell Signaling, 1:50); followed by peroxidase-conjugated secondary antibody. Normal kidney served as positive controls for IGF-1, IGF-1R, and IGF-2. Negative controls included omission of primary antibody.

All immunohistochemistry evaluation was performed in a blinded manner by an attending pathologist. The slides were scored semiquantitatively. A score of 0 indicated no staining, 1 indicated focal weak staining, 2 indicated moderately positive staining, and 3 indicated intensely positive staining. Tumors with strong staining (scores 2 to 3) were grouped, and tumors with weak or no staining (scores 0 to 1) were grouped. All analyses were performed in triplicate.

### Correlation with clinicopathologic factors

Electronic medical records of 27 patients who underwent liver resection for HCC between 1994 and 2011 were reviewed. Patients were diagnosed with NAFLD-associated HCC if they did not have a history of alcohol abuse or viral hepatitis, had features of the metabolic syndrome, such as diabetes, hyperlipidemia, or hypertension, and had evidence of hepatic steatosis on pathologic examination. Tumors were staged after resection according to the 7th edition of the *American Joint Committee on Cancer Staging Manual*[16]. This study was approved by the Fox Chase Cancer Center Institutional Review Board, which waived the requirement for informed consent.

### Statistical analysis

Categorical variables were compared using Fisher’s exact test. Continuous data were expressed as the median and range and compared using the Mann-Whitney test. Survival analysis was performed using the Kaplan-Meier method, and differences in survival were compared using the log-rank test. Factors significant in univariate analysis (*P* < 0.05) were entered into multivariate analysis, performed using the Cox proportional-hazards model. All statistical tests were two-sided; the significance parameter was set at *P* < 0.05. Statistical analysis was performed using SPSS software, version 17.0 (SPSS, Inc., Chicago, IL, USA).

## Results

Clinicopathologic factors of the 27 patients who underwent resection of HCC are depicted in Table 1. Median follow-up was 26 months (range, 2 to 136 months). One patient died from postoperative complications.

**Table 1:** Clinicopathologic factors in 27 patients undergoing resection of hepatocellular carcinoma

**Clinicopathologic factor**	***n***
Median age (range)	74 (34 to 81)
Male	14
*Race*	
White	24
Asian	2
African American	1
*Risk factor for hepatocellular carcinoma*	
Nonalcoholic fatty liver disease	13
Hepatitis C	7
Alcoholic hepatitis	2
Transformed adenoma	2
Other^a^	3
*Cirrhosis*	
Yes	10
No	17
*Vascular invasion*	
Yes	9
No	18
*Multiple tumors*	
Yes	5
No	22
*Maximum tumor size > 5 cm*	
Yes	14
No	13
*Preoperative median serum α-fetoprotein level (range)*	6 (1 to 8122)
*Preoperative median Model for End-Stage Liver Disease score (range)*	7 (6 to 12)
*Tumor grade*	
Well differentiated	13
Moderately differentiated	8
Poorly differentiated	4
Unknown	2
*Hepatectomy*	
Minor (resection of <3 segments)	22
Major (resection of ≥3 segments)	5
*American Joint Committee on Cancer, 7th edition stage*	
I	16
II	8
IIIA	2
IIIB	1

^a^Other diagnoses were fibrolamellar carcinoma, clear cell carcinoma, and hemochromatosis.

Expression levels of IGFs and their receptors were not associated with patients’ underlying liver disease, with the exception of IGF-1R (Tables 2 and 3). In HCC tumor samples, IGF-1R expression was low in 24 of 27 patients. In adjacent noncancerous liver, IGF-1R was strongly expressed in only one patient with alcoholic hepatitis. Intense immunoreactivity was observed for IGF-2 in all paired samples of tumor and background liver.

**Table 2:** Proportion of patients with positive immunohistochemical staining in hepatocellular carcinoma tumor tissues stratified by underlying liver disease

	**Nonalcoholic fatty liver disease**	**Hepatitis C**	**Alcoholic hepatitis**	**Transformed adenoma**	**Other**^**a**^	***P***
IGF-1	8/13	6/7	1/2	2/2	3/3	0.418
IGF-1R	0/13	1/7	1/2	1/2	0/3	0.085
IGF-2	13/13	7/7	2/2	2/2	3/3	Not applicable
IGF-2R	6/13	3/7	1/2	1/2	2/3	0.972

^a^One patient each with fibrolamellar carcinoma, clear cell carcinoma, and hemochromatosis. Positive staining was defined as moderately or intensely positive staining.

**Table 3:** Proportion of patients with positive immunohistochemical staining in adjacent non-neoplastic liver stratified by underlying liver disease

	**Nonalcoholic fatty liver disease**	**Hepatitis C**	**Alcoholic hepatitis**	**Transformed adenoma**	**Other**^**a**^	***P***
IGF-1	8/13	4/7	2/2	2/2	3/3	0.403
IGF-1R	0/13	0/7	1/2	0/2	0/3	0.011
IGF-2	13/13	7/7	2/2	2/2	3/3	Not applicable
IGF-2R	6/13	2/7	2/2	2/2	3/3	0.087

^a^One patient each with fibrolamellar carcinoma, clear cell carcinoma, and hemochromatosis. Positive staining was defined as moderately or intensely positive staining.

### Associations between clinicopathologic factors and expression of IGF ligands and receptors in HCC tumor samples

The HCC expression of IGF-2R was significantly higher in men, with strong staining in 10 of 14 men versus 3 of 13 women (*P* = 0.021). Expression levels of IGFs and their receptors in HCC were not associated with patient age, American Joint Committee on Cancer stage, tumor size, number, grade, serum α-fetoprotein concentration, Model for End-Stage Liver Disease score, or the presence of vascular invasion (all *P* > 0.05).

### Associations between clinicopathologic factors and expression of IGF ligands and receptors in paired samples of adjacent non-neoplastic liver

**Figure 2:**
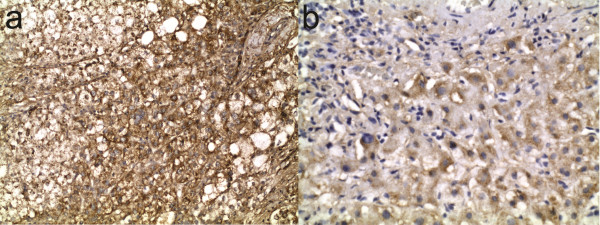
**Representative IGF-1 expression in hepatocellular carcinoma.** Immunohistochemical staining in a patient with cirrhosis showing higher IGF-1 expression in **(a)** hepatocellular carcinoma compared with **(b)** adjacent non-neoplastic liver. Original magnifications, 20× and 40× for **(a)** and **(b)**, respectively.

Expression of IGF-1 was low in non-neoplastic liver in 6 of 10 patients with cirrhosis, compared with 2 of 17 patients without cirrhosis (*P* = 0.025, Figure 2).

Expression of IGFs and their receptors in adjacent noncancerous liver was not associated with patient age, sex, American Joint Committee on Cancer stage, tumor size, number, grade, serum α-fetoprotein, or the presence of vascular invasion (all *P* > 0.05).

### Overall survival and differential IGF-1 expression in tumor versus adjacent non-neoplastic liver

**Figure 3:**
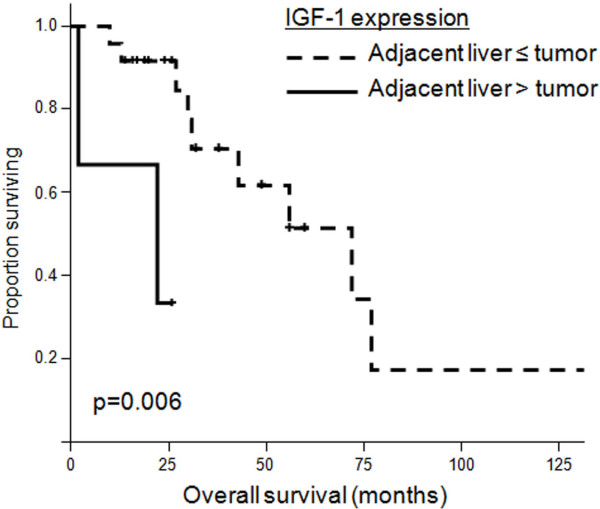
**Kaplan-Meier curves of patients with hepatocellular carcinoma.** Overall survival in 27 patients undergoing resection of hepatocellular carcinoma was significantly lower in patients with higher IGF-1 expression in adjacent non-neoplastic liver compared with tumor.

Higher IGF-1 expression in non-neoplastic liver was associated with significantly poorer median survival of 22 months (95% confidence interval, 0 to 54 months), compared with 72 months (95% confidence interval, 37 to 107 months) with equal or lower IGF-1 expression in adjacent liver relative to tumor (*P* = 0.006, Figure 3). On multivariate analysis, higher IGF-1 expression in adjacent noncancerous liver remained an independent predictor of overall survival (Table 4).

**Table 4:** Multivariate analysis of overall survival

**Factor**	**Median overall survival (range)**^**a**^	**Univariate *P *** **value**	**Multivariate *P *** **value**	**Hazard ratio (95% confidence ratio)**
Cirrhosis		0.506		
Yes, *n* = 10	56 (29 to 83)			
No, *n* = 17	77 (27 to 127)			
Vascular invasion		0.020	0.048	4.477 (1.013 to 19.789)
Yes, *n* = 9	30 (22 to 38)			
No, *n* = 18	72 (43 to 101)			
Multiple tumors		0.469		
Yes, *n* = 5	56 (35 to 77)			
No, *n* = 22	72 (12 to 132)			
Size >5 cm		0.448		
Yes, *n* = 14	31 (0 to 73)			
No, *n* = 13	72			
Underlying liver disease		0.652		
Nonalcoholic fatty liver disease, *n* = 13	30			
Hepatitis C, *n* = 7	77			
Alcoholic hepatitis, *n* = 2	22			
Transformed adenoma, *n* = 2	Not reached			
Other, *n* = 3^b^	31			
Sex		0.275		
Male, *n* = 14	72 (35 to 109)			
Female, *n* = 13	43 (26 to 60)			
Age	Not estimable	0.099		
American Joint Committee on Cancer stage		0.212		
I, *n* = 16	72 (8 to 136)			
II, *n* = 8	31 (29 to 33)			
III, *n* = 3	136			
Grade		0.427		
1, *n* = 13	72 (43 to 102)			
2, *n* = 8	30			
3, *n* = 4	Not estimable			
Unknown, *n* = 2	13			
Serum α-fetoprotein concentration	Not estimable	0.099		
Model for End-Stage Liver Disease score	Not estimable	0.482		
Higher IGF-1 expression in adjacent liver than tumor		0.006	0.040	7.889 (1.096 to 56.761)
Yes, *n* = 3	22 (0 to 54)			
No, *n* = 24	72 (37 to 107)			

^a^Range not estimable in all categories. ^b^One patient each with fibrolamellar carcinoma, clear cell carcinoma, and hemochromatosis.

## Discussion

Nonalcoholic fatty liver disease is emerging as a major public health concern in the United States, owing to its progression to steatohepatitis, cirrhosis, and HCC. It affects 30% of the United States population and 90% of the morbidly obese [1]. The molecular mechanisms underlying malignant transformation in NAFLD to HCC are not well elucidated. Dysregulation of the IGF pathway is implicated in the pathogenesis of HCC, particularly in patients with NAFLD and the metabolic syndrome, which are associated with insulin resistance, chronic hyperinsulinemia, and increased levels of bioavailable IGF-1. In this study, expression levels of IGF-1, IGF-2, and their receptors were determined in paired samples of tumor and adjacent non-neoplastic liver from patients who underwent liver resection for HCC. The most common chronic liver disease was NAFLD, followed by hepatitis C and alcoholic hepatitis. Our preliminary results demonstrate that expression levels of IGF ligands and receptors are not associated with patients’ underlying liver disease.

In this report, IGF-1 expression in adjacent non-neoplastic liver was low in patients with cirrhosis compared with noncirrhotic patients. These results are consistent with prior studies showing reduced serum levels of IGF-1 in patients with hepatocellular dysfunction [17,18]. The chief stimulus for IGF-1 synthesis is growth hormone binding to its receptors on hepatocytes, leading to *Igf1* gene transcription. In patients with chronic liver disease, the decline in circulating IGF-1 levels is hypothesized to result from decreased functional liver mass and an attenuated IGF-1 response to growth hormone stimulation. In a recent study of 288 patients with HCC, low plasma IGF-1 levels were found to correlate with advanced clinicopathologic parameters and poor overall survival [19].

A key finding in this study was that higher IGF-1 expression in adjacent non-neoplastic liver than in tumor correlated with significantly poorer survival after resection of HCC. On multivariate analysis, higher IGF-1 in background liver and vascular invasion remained independent predictors of worse outcome. Activation of the IGF-1 signaling cascade leads to upregulation of downstream mitogens, including MAPK and PI3K/Akt. Preclinical studies have identified local IGF-1 synthesis in several cancer cell types, including HCC, leading to autocrine and paracrine effects on cell cycle progression and inhibition of apoptosis [8,12]. Our results support the hypothesis that during hepatocarcinogenesis, secretion of IGF-1 by adjacent hepatocytes may lead to paracrine stimulation of HCC and more aggressive tumor behavior.

The present data underscore the importance of IGF-2 in the pathogenesis of HCC. We found strong IGF-2 immunoreactivity in all samples of HCC and adjacent non-neoplastic liver. Other investigators have observed upregulation of IGF-2 in 16% to 100% of HCCs [20,21]. Consistent with our findings, Park *et al.*[22] identified IGF-2 immunoreactivity in paired noncancerous liver tissues, both at a distance from HCC tumors and in close contact with the growing edges of tumor. In normal adult liver, IGF-2 expression is low, and its physiologic function is unclear. However, in hepatitis and cirrhosis, IGF-2 expression is increased, owing to reactivation of fetal promoters that induce *Igf2* gene transcription [23]. Upregulation of IGF-2 leads to multiple oncogenic effects, including cell proliferation, anti-apoptosis, and angiogenesis [3].

The chief limitation of this study is the small numbers of patients, and our results require validation in a larger sample population. We did not examine immunoreactivity of insulin receptor substrates and binding proteins, which are key components of the IGF signaling cascade. In addition, we plan to examine downstream effects of IGF signaling dysregulation, including effects on the PI3K and MAPK pathways.

## Conclusions

In conclusion, NAFLD was the most common underlying liver disease in this study of patients undergoing resection of HCC. Expression levels of IGF ligands and receptors did not correlate with patients’ underlying liver disease. Others have reported overexpression of the IGF receptors in HCC, which was not observed in the current study, although our results should be interpreted cautiously, owing to the small number of patients [23]. Insulin-like growth factor-2 demonstrated strong immunoreactivity in all patient samples of tumor and adjacent liver, indicating the importance of IGF-2 in early and late stages hepatocarcinogenesis. Insulin-like growth factor-1 staining was low in the background liver of cirrhotic patients, consistent with previous literature showing low IGF-1 levels in patients with hepatocellular dysfunction. Patients with higher expression of IGF-1 in adjacent liver than in tumor had significantly lower overall survival, suggesting that increased IGF-1 synthesis by neighboring hepatocytes may lead to aggressive tumor biology. These results merit confirmation in a larger number of patients and support continued investigation of the IGF signaling cascade for potential therapeutic targets in HCC.

## Abbreviations

HCC: hepatocellular carcinoma; IGF: insulin-like growth factor; IGF-1: insulin-like growth factor-1; IGF-1R: insulin-like growth factor-1 receptor; IGF-2: insulin-like growth factor-2; IGF-2R: insulin-like growth factor-2 receptor; MAPK: mitogen-activated protein kinase; NAFLD: nonalcoholic fatty liver disease; PI3K/Akt: phosphatidylinositide 3-kinase/Akt; SHC: Src homology 2 domain-containing transforming protein 1.

## Competing interests

The authors declare that they have no competing interests.

## Authors’ contributions

YSC participated in the study design, analyzed the data, and prepared the manuscript. MH acquired materials, interpreted data, and prepared the manuscript. LR acquired materials, performed the immunohistochemistry, and prepared the manuscript. MVM participated in the study design and prepared the manuscript. All authors read and approved the final manuscript.
